# Pharmacogenetic Study of Drug-Metabolising Enzyme Polymorphisms on the Risk of Anti-Tuberculosis Drug-Induced Liver Injury: A Meta-Analysis

**DOI:** 10.1371/journal.pone.0047769

**Published:** 2012-10-17

**Authors:** Yu Cai, JiaYong Yi, ChaoHui Zhou, XiZhong Shen

**Affiliations:** 1 Department of Gastroenterology, Zhongshan Hospital, Fudan Unversity, Shanghai, People’s Republic of China; 2 Departments of Orthopedics, Zhongshan Hospital, Fudan Unversity, Shanghai, People’s Republic of China; IPO, Inst Port Oncology, Portugal

## Abstract

**Background:**

Three first-line antituberculosis drugs, isoniazid, rifampicin and pyrazinamide, may induce liver injury, especially isoniazid. This antituberculosis drug-induced liver injury (ATLI) ranges from a mild to severe form, and the associated mortality cases are not rare. In the past decade, many investigations have focused the association between drug-metabolising enzyme (DME) gene polymorphisms and risk for ATLI; however, these studies have yielded contradictory results.

**Methods:**

PubMed, EMBASE, ISI web of science and the Chinese National Knowledge Infrastructure databases were systematically searched to identify relevant studies. A meta-analysis was performed to examine the association between polymorphisms from 4 DME genes (NAT2, CYP2E1, GSTM1 and GSTT1) and susceptibility to ATLI. Odds ratios (ORs) and 95% confidence intervals (CIs) were calculated. Heterogeneity among articles and their publication bias were also tested.

**Results:**

38 studies involving 2,225 patients and 4,906 controls were included. Overall, significantly increased ATLI risk was associated with slow NAT2 genotype and GSTM1 null genotype when all studies were pooled into the meta-analysis. Significantly increased risk was also found for CYP2E1*1A in East Asians when stratified by ethnicity. However, no significant results were observed for GSTT1.

**Conclusions:**

Our results demonstrated that slow NAT2 genotype, CYP2E1*1A and GSTM1 null have a modest effect on genetic susceptibility to ATLI.

## Introduction

Tuberculosis (TB) is an important infectious disease plaguing the developed and developing countries worldwide, with more than nine million new cases of active tuberculosis reported annually. Isoniazid (INH), rifampicin (RIF) and pyrazinamide (PZA) are all widely used as first-line multidrug therapy for TB. Anti-tuberculosis drug induced liver injury (ATLI), a common serious adverse drug reaction, is one of the most challenging clinical problems, cause of hospitalization and life-threatening events. ATLI can be fatal if therapy is not interrupted on time, and the subsequent adherence problem may cause treatment failure and, relapse or drug resistance [1], [2].

Of the various antituberculosis regimens, isoniazid is the main drug to induce hepatotoxicity [3]. Metabolic intermediates of isoniazid are incriminated to be the cause of hepatotoxicity. In the liver, isoniazid is first metabolized into acetylisoniazid via N-acetyltransferase (NAT) [4], followed by hydrolysis to acetylhydrazine. Acetylhydrazine is proposed to be oxidized into hepatotoxic intermediates by cytochrome P450 2E1 (CYP2E1) [5] and some hepatotoxic intermediates can be detoxified by glutathione S-transferase (GST) enzyme [6]. Drug-metabolizing enzymes (DME) have critical effects on both the synthesis and detoxification of reactive metabolites [7]. Therefore, studies on genetic predisposition for anti-TB drug-induced liver injury have focused on a few metabolizing enzymes including N-acetyltransferase 2 (NAT2), CYP2E1, GSTM1 and GSTT1.

Over the past few years, considerable efforts have been devoted to exploring the relationships between the DME polymorphisms and ATLI among various populations. However, existing studies have yielded inconsistent results. These disparate findings may be due partly to insufficient power, false-positive results, and publication biases. Therefore, we performed a meta-analysis of the published studies to clarify this inconsistency and obtain summary risk estimates for the association of specific polymorphism in DME and risk of ATLI.

## Materials and Methods

### Literature Search Strategy

Eligible literature published before the end of May 2012 were identified through a search of PubMed, EMBASE, ISI Web of Science and CNKI (Chinese National Knowledge Infrastructure) without language restriction. Search term combinations were as follows: drug-metabolising enzymes, antitubercular agents, drug-induced hepatotoxicity, drug-induced liver injury, genetic polymorphism, arylamine N-acetyltransferase, glutathione-S-transferase, cytochrome 2E1, acetylator phenotype, genetic susceptibility. All reference lists from the main reports and relevant reviews were hand searched for additional eligible studies.

### Eligible Studies and Data Extraction

Eligible studies had to meet all of the following criteria: (i) the studies were published in peer-reviewed journals and were independent studies using original data; (ii) the studies provided genotype distribution information of polymorphism in both cases and controls or odds ratio (OR) with its 95% confidence interval and P value; (iii) the studies investigated the DME polymorphism using either case-control or cohort design; (iv) the studies described the genotyping method, equipment, and protocols used or provided reference to them and (v) the studies used TB patients without ATLI as controls.

For each study, the following data were extracted independently by two authors: first author’s surname, year of publication, diagnosis criterion, age, sex, ethnicity, genotyping method, total number of cases and controls and genotype frequency in cases and controls. The results were compared, and disagreements were discussed and resolved with consensus.

### Statistical Methods

The proposed risk genotypes are NAT2 slow acetylator (without wild-type NAT2*4 allele), CYP2E1*1A/*1A (homozygous wild type c1/c1) and homozygous null GST genotype (GSTM1 null/null or GSTT1 null/null). Our primary analysis for measuring the overall effects of every DME was to compare the genotype distribution for ATLI patients against controls by the contrast of the above risk genotypes vs. other combined genotypes.

Odds ratio (OR) with 95% confidence intervals (CIs) was used to assess the strength of association between gene polymorphism and ATLI risk. Cochran’s chi-square-based Q statistic test was performed in order to assess possible heterogeneity between the individual studies and thus to ensure that each group of studies was suitable for meta-analysis. ORs were pooled according to the method of DerSimonian and Laird that takes into account the variation between studies, and 95% CI were constructed using Woolf’s method [8], [9]. The Z test was used to determine the significance of the pooled OR. Sensitivity analyses were performed to assess the stability of the results, namely, a single study in the meta-analysis was deleted each time to reflect the influence of the individual data set to the overall OR. Publication bias was assessed using Egger’s test [10] and Begg’s funnel plots [11]. All P values are two-sided, and P<0.05 were considered statistically significant. Statistical analyses were done with Stata (version 10.0).

## Results

### Characteristics of Studies

The combined search yielded 412 references. Study selection process was shown in Figure S1. A total of 38 studies were finally included with 2,225 patients and 4,906 controls [12]–[42]. For the CYP2E1, 13 studies were available, including a total of 674 cases and 1,990 controls. For the NAT2, 24 studies involved a total of 1,116 cases and 2,655 controls. For the GST M1, 11 studies involved a total of 896 cases and 1,604 controls. For the GST T1, 10 studies involved a total of 792 cases and 1,493 controls. Of the cases, 80% were East Asian, 8% were Indian, 7% were Caucasian, and 5% were of other ethnic populations. The detailed characteristics of the studies included in this meta-analysis are shown in Table 1.

**Table 1:** Characteristics of the studies included in the meta-analysis.

Study	Year	Ethnicity	Gene	No. of case/control	Mean age of case/control	Gender component in caseand control (% male)	Genotyping method
Tang [12]	2012	Chinese	CYP2E1; GSTM1; GSTT1	89/356	32.7/43.6	73.0/73.0	RFLP
An [13]	2012	Chinese	NAT 2; CYP2E1	101/107	36.0/33.4	55.4/70.0	Sequencing
Rana [14]	2012	Indian	NAT2	50/201	45.3/43.8	24.8/75.2	RFLP
Mahmoud [15]	2011	Tunisian	NAT2	14/52	42.4/42.1	42.8/48.1	RFLP
Zhu [16]	2011	Chinese	GSTM1; GSTT1	228/300	39.8/37.5	57.9/53.3	PCR
Fernandez [17]	2011	Spanish	NAT2	50/67	34/30.5	54.0/56.7	RFLP
Sotsuka [18]	2011	Japanese	NAT2; CYP2E1; GSTM1; GSTT1	52/92	54.9/50.4	92.3/73.9	RFLP; PCR
Sistanizad [19]	2011	Iranian	NAT2	14/36	43.1/49.6	57.1/55.5	RFLP
Huang [20]	2011	Chinese	NAT2	119/198	41.0/44.0	66.4/64.1	Sequencing
Teixeira [21]	2011	Brazilian	CYP2E1; GSTM1; GSTT1	26/141	47.6/43.0	61.5/52.5	Sequencing; PCR
Yimer [22]	2011	European	NAT2	41/160	NA/NA	NA/NA	Sequencing
Bose [23]	2011	Indian	NAT2; CYP2E1	41/177	38.0/36.0	43.9/47.4	RFLP
Chatterjee [24]	2010	Indian	GSTM1; GSTT1	51/100	37.2/33.2	49.0/63.0	PCR
Wang [25]	2010	Chinese	GSTM1; CYP2E1	104/111	48.6/44.7	67.3/67.6	PCR
Lee [26]	2010	Chinese	NAT2; CYP2E1	45/95	58.4/54.9	60.0/66.3	Taqman
Kim [27]	2010	Korean	GSTM1; GSTT1	57/190	47.3/42.4	59.6/67.9	PCR
Wu [28]	2010	Chinese	NAT2	155/162	NA/NA	58.0/60.0	Taqman
Guo [29]	2010	Chinese	NAT2	106/106	48.8/48.6	68.9/68.9	RFLP
Chen [30]	2010	Chinese	CYP2E1	103/236	45.0/45.7	78.6/66.1	RFLP
Wang [31]	2009	Chinese	CYP2E1	104/111	47.7/45.5	61.1/64.0	RFLP
Guo [32]	2009	Chinese	GSTM1; GSTT1	106/106	48.8/48.6	68.9/68.9	PCR
Kim [33]	2009	Korean	NAT2	67/159	42.1/42.8	65.7/65.4	SNPstream
Yamada [34]	2009	Japanese	NAT2; CYP2E1	23/147	NA/NA	13.0/42.8	Sequencing, RFLP
Wang [35]	2009	Chinese	NAT2	36/36	NA/NA	NA/NA	RFLP
Leiro [36]	2008	Spanish	GSTM1; GSTT1	35/60	34.0/31.0	40.0/41.7	PCR
Bozok [37]	2008	Turkish	NAT2	30/70	39.8/37.3	50.0/72.8	PCR
Possuelo [38]	2008	Brazilian	NAT2	14/240	38.9/36.5	50.0/67.9	Sequencing
Huang [39]	2007	Chinese	GSTM1; GSTT1	115/115	60.3/59.1	63.5/63.5	PCR
Cho [40]	2007	Korean	NAT2; CYP2E1	18/114	51.2/46.7	66.7/55.3	Sequencing
Higuchi [41]	2007	Japanese	NAT2	18/82	60.8/64.7	50.0/57.3	RFLP
Vuilleumier [42]	2006	Swiss	CYP2E1	34/55	NA/NA	NA/NA	RFLP
Shimizu [43]	2006	Japanese	NAT2	10/32	60.5/64.9	70.0/46.9	RFLP
Roy [44]	2006	Indian	CYP2E1	8/101	NA/NA	NA/NA	RFLP
Wang [45]	2004	Chinese	NAT2	32/35	NA/NA	53.1/45.7	RFLP
Huang [46]	2003	Chinese	NAT2; CYP2E1	49/269	70.0/59.0	18.4/14.9	RFLP
Huang [47]	2002	Chinese	NAT2	33/191	73.3/63.7	87.9/88.5	RFLP
Roy [48]	2001	Indian	NAT2; GSTM1; GSTT1	33/33	NA/NA	NA/NA	PCR
Ohno [49]	2000	Japanese	NAT2	14/63	NA/NA	NA/NA	RFLP

### Association of CYP2E1 Gene with ATLI

**Figure 1:**
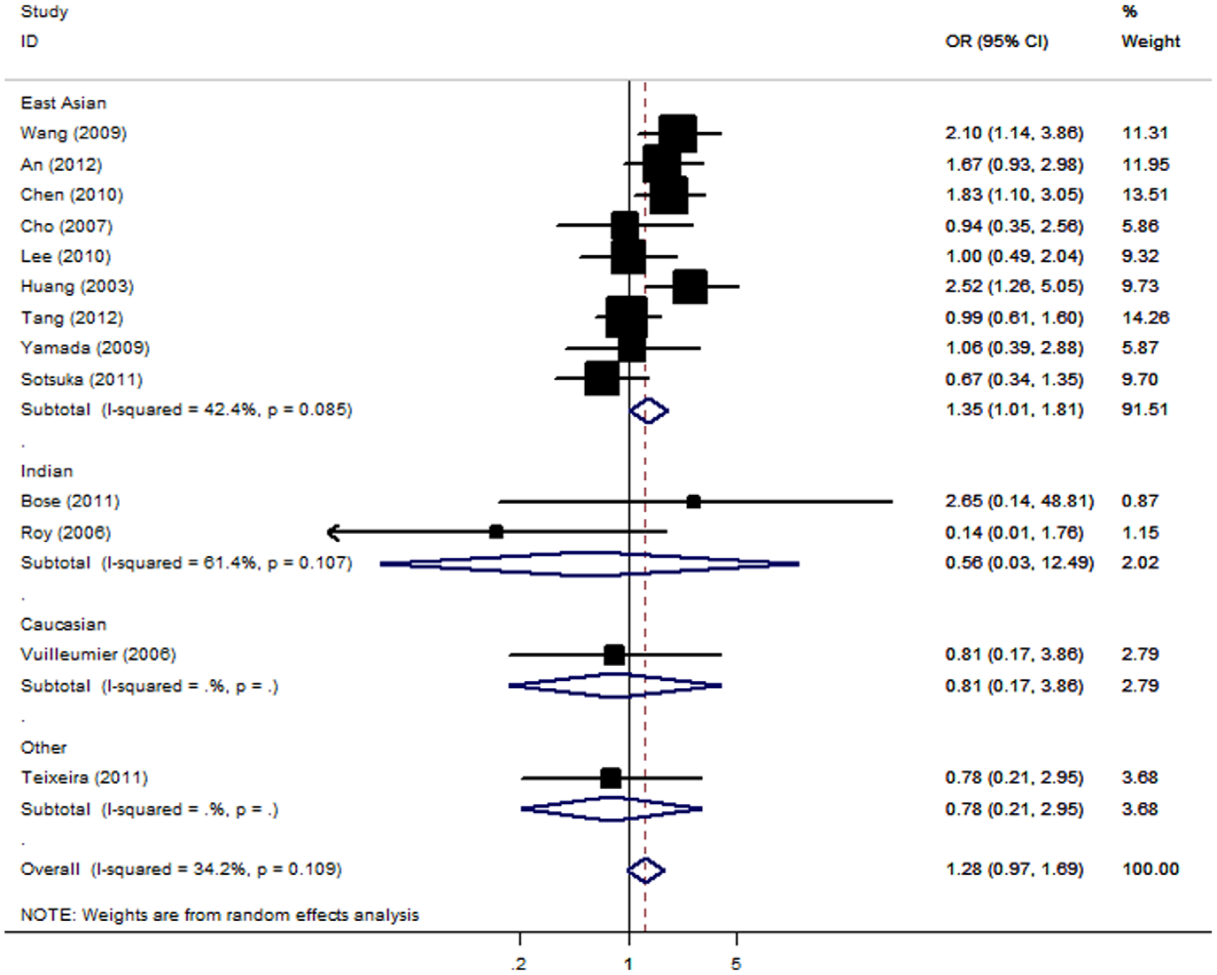
Forest plot from the meta-analysis of ATLI and *CYP2E1*.

Meta-analysis revealed no statistically significant association between ATLI and CYP2E1 c1/c1 genotype [OR = 1.28; 95% CI: 0.97–1.69; *P(Z) = *0.08; *P(Q)* = 0.11]. When studies were stratified for ethnicity, significant risks were found among East Asians [OR = 1.35; 95% CI: 1.01–1.81; *P(Z)* = 0.04; *P(Q)* = 0.08]. However, no significant associations were detected among Indian, Caucasian and other ethnic populations (Figure 1). In the subgroup analyses by sample size, the summary OR for big studies of the c1/c1 genotype was 1.36 [95% CI: 0.92–2.00; *P(Z) = *0.12; *P(Q)* = 0.05] and for small studies was 1.17 [95% CI: 0.75–1.81; *P(Z) = *0.49; *P(Q)* = 0.28].

### Association of NAT2 Gene with ATLI

**Figure 2:**
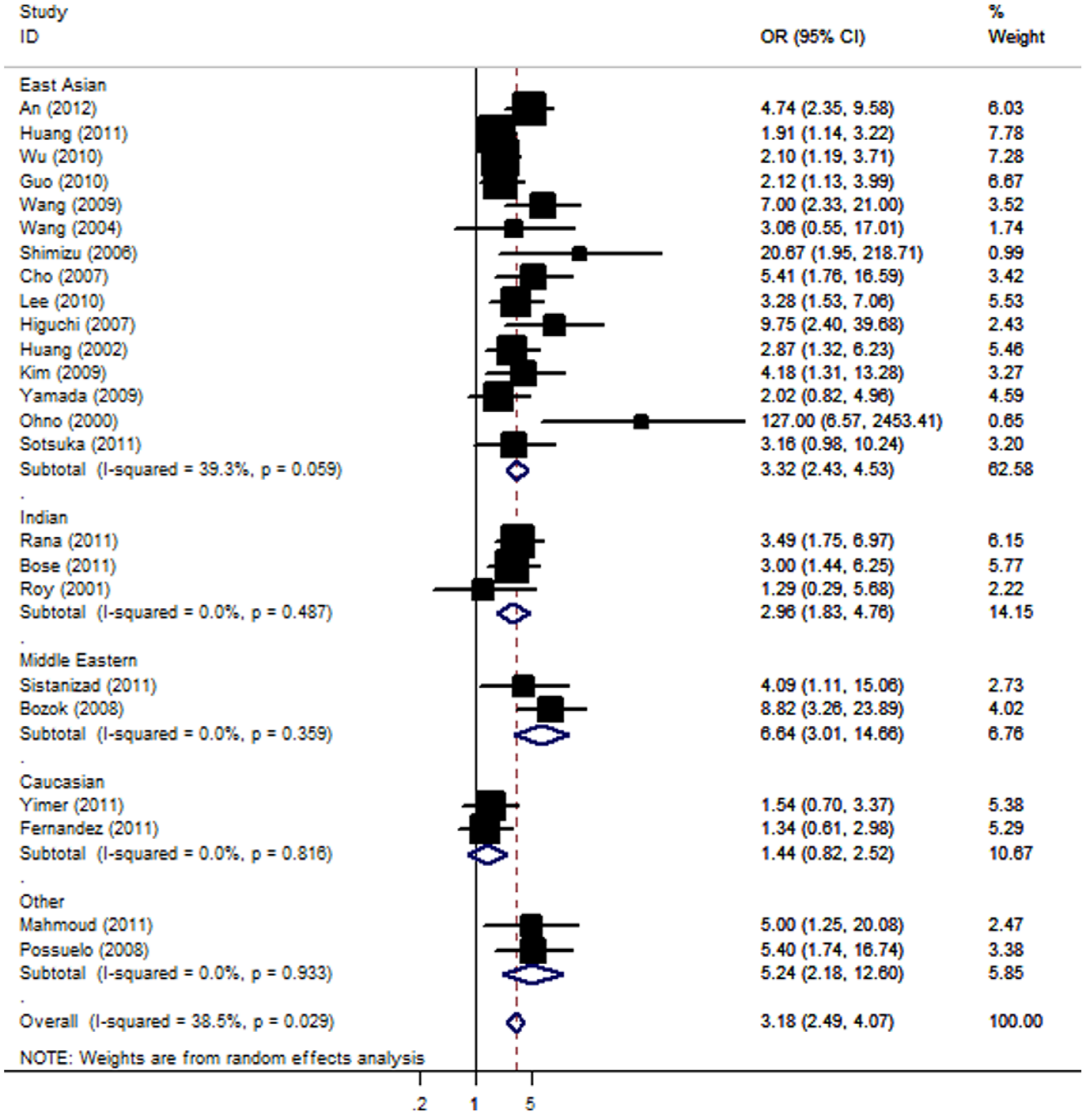
Forest plot from the meta-analysis of ATLI and *NAT2*.

Overall, there was evidence of an association between the increased risk of ATLI and the variant when all eligible studies were pooled into the meta-analysis. Using random effect model, the summary OR of the NAT2 slow acetylator genotype for ATLI was 3.18 [95% CI: 2.49–4.07; *P(Z)* <10^−5^; *P(Q)* = 0.03]. When stratifying for ethnicity, an OR of 3.32 [95% CI: 2.43–4.53; *P(Z)* <10^−5^; *P(Q)* = 0.06], 2.96 [95% CI: 1.83–4.76; *P(Z)* <10^−4^; *P(Q)* = 0.49], 6.64 [95% CI: 3.01–14.66; *P(Z)* <10^−4^; *P(Q)* = 0.36] and 5.24 [95% CI: 2.18–12.60; *P(Z)* <10^−4^; *P(Q)* = 0.93] resulted for the slow acetylator genotype, among East Asian, Indian, Middle Eastern and other ethnic population, respectively (Figure 2). Unfortunately, we failed to detect any association to ATLI risk for Caucasians. Subsidiary analyses of control source yielded an overall OR for big studies of 2.48 [95% CI: 1.87–3.30; *P(Z)* <10^−5^; *P(Q)* = 0.26] and for small studies of 3.92 [95% CI: 2.75–5.58; *P(Z)* <10^−5^; *P(Q)* = 0.06].

### Association of GSTM1 and GSTT1 Gene with ATLI

**Figure 3:**
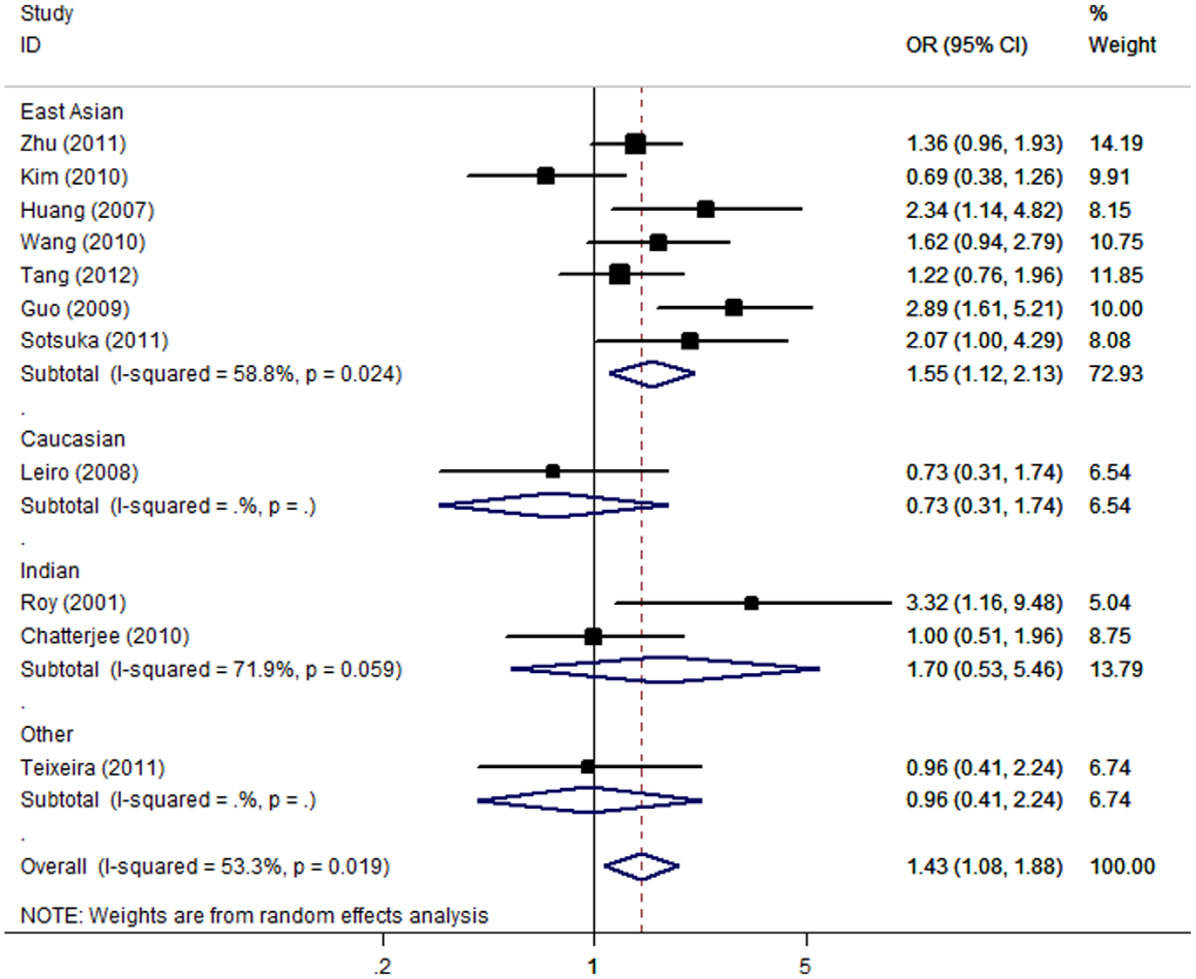
Forest plot from the meta-analysis of ATLI and *GST M1*.

For ATLI risk and the null genotype of GSTM1, our meta-analysis gave an overall OR of 1.43 [95% CI: 1.08–1.88; *P(Z) = *0.01; *P(Q) = *0.02] with statistically significant between-study heterogeneity. This analysis is based on pooling of data from a number of different ethnic populations. When stratifying for ethnicity, an OR of 1.55 [95% CI: 1.12–2.13; *P(Z)* = 0.008; *P(Q)* = 0.02] resulted for null genotype among East Asians; while no significant association was found among Caucasian and Indian populations (Figure 3). By considering sample size subgroups, the OR was 1.61 [95% CI: 1.18–2.19; *P(Z) = *0.003; *P(Q)* = 0.005] in big studies, compared to 1.26 [95% CI: 0.54–2.95; *P(Z) = *0.59; *P(Q)* = 0.08] in small studies.

**Figure 4:**
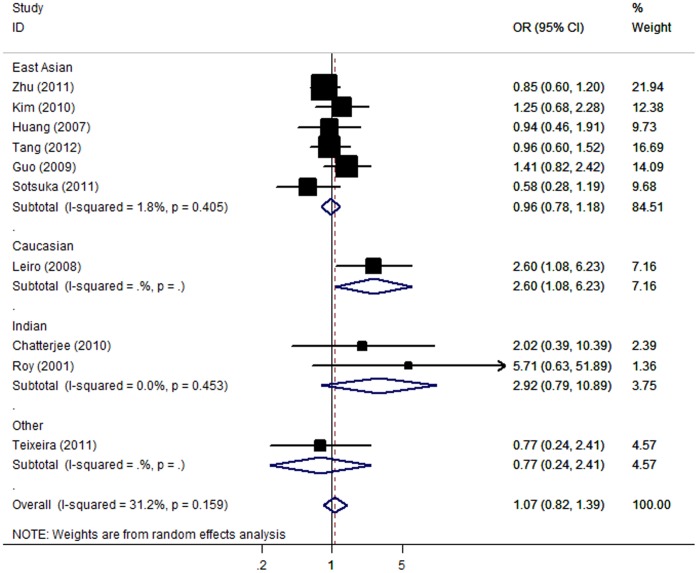
Forest plot from the meta-analysis of ATLI and *GST T1*.

The meta-analysis resulted in a statistically non-significant association between GSTT1 deficiency and ATLI. The overall OR was 1.07 [95% CI: 0.82–1.39; *P(Z) = *0.61; *P(Q)* = 0.16]. When stratifying for ethnicity, an OR of 0.96 [95% CI: 0.78–1.18; *P(Z) = *0.69; *P(Q)* = 0.40] and 2.92 [95% CI: 0.79–10.89; *P(Z)* = 0.11; *P(Q)* = 0.45] resulted for null genotype, among East Asian and Indian populations, respectively (Figure 4). No significant association was found in stratified analyses according to sample size. The OR was 0.97 [95% CI: 0.79–1.19; *P(Z) = *0.76; *P(Q)* = 0.44] in big studies, 1.88 [95% CI: 0.68–5.20; *P(Z)* = 0.23; *P(Q)* = 0.15] in small studies.

The effect of each genotype of GSTs was independently assessed. The data on both null genotype of GSTs among cases and controls were available in five studies, which included 460 cases and 1006 controls. The interaction between GSTM1 null and GSTT1 null, for which an OR of 1.12 [95% CI: 0.86–1.48; *P(Z) = *0.40; *P(Q)* = 0.69] for ATLI appeared in compared with individuals with the positive genotypes.

### Sensitivity Analyses and Publication Bias

Sensitivity analyses were performed to assess the influence of each individual study on the pooled OR by sequential removal of individual studies. The results suggested that no individual study significantly affected the pooled OR, thus suggesting that the results of this meta-analysis are stable.

A funnel plot of these 24 included studies concerning NAT2 and ATLI suggested a possibility of the preferential publication of positive findings in smaller studies (t = 3.72, *P* = 0.001; Figure S2). The shape of the funnel plots was symmetrical (Figures S3, S4 and S5) for CYP2E1, GSTM1 and GSTT1. The statistical results still did not show publication bias in these studies for CYP2E1 (t = −1.27, *P* = 0.23), GSTM1 (t = 0.32, *P* = 0.76) and GSTT1 polymorphism (t = 1.65, *P* = 0.14).

## Discussion

This is the most comprehensive meta-analysis concerning the relationship between polymorphisms of three DME genes and ATLI risk. Its strength was based on the accumulation of published data giving greater information to detect significant differences. In total, the meta-analysis involved 38 studies for ATLI that provided 2,225 patients and 4,906 controls. Our results demonstrated that the slow NAT2 genotype and GSTT1 null polymorphism is a risk factor for developing ATLI. Although no significant association between CYP2E1 *1A polymorphism and ATLI susceptibility was detected in the overall comparison, we found that the CYP2E1 *1A polymorphism was associated with increased risk of ATLI among East Asian populations when stratified by ethnicity. Ethnic differences may contribute to these different results, since the CYP2E1 c2 allele distribution of the polymorphism varies between East Asian and other ethnic populations [50]. However, we failed to detect any positive relationship between ATLI and GSTT1 null polymorphism.

As the pathogenic mechanism of ATLI is poorly understood, most studies were based on INH metabolic pathway. For predicting ATLI, the NAT2 genotype is seemingly more important than other genotypes, because this genotype possesses high susceptibility to ATLI with OR of 3.18 compared with CYP2E1 (OR = 1.28) and GSTM1 (OR = 1.43). Slow acetylators not only acetylate isoniazid more slowly but also monoacetylhydrazine, the immediate precursor of the toxic intermediates, to the harmless diacetylhydrazine [51]. This protective acetylation is further suppressed by isoniazid. Therefore, slow acetylators may critically increase the accumulation of toxic metabolites indirectly. The action of NAT in the disposition of isoniazid is followed by CYP2E1. Earlier reports demonstrated CYP2E1 activity was less inhibited by isoniazid in subjects with CYP2E1 *1A/*1A genotype than in those with other genotypes [46]. Therefore, under the administration of isoniazid, subjects with CYP2E1 *1A/*1A genotype have higher CYP2E1 activity than those with other genotypes, and, hence, may produce more hepatotoxins and finally increase the risk of liver injury.

GST, as an important phase II detoxification enzyme, was correlated to the susceptibility of alcoholic liver disease and many cancers [6]. Subjects with homozygous ‘null’ mutant genotype of GSTM1 or GSTT1 have been found to lose enzymatic activity [6]. It is speculated that people with null GSTM1 or GSTT1 genotypes could not detoxify the toxic reactive metabolites efficiently, and thus have higher risk of drug-induced liver injury and many cancers. However, failing to identify the association between GSTT1 polymorphism and ATLI may be due partly to the small sample size and low frequency of patients with ATLI; additional studies with large sample size are therefore needed to confirm our finds.

In interpreting the results, some limitations of this meta-analysis should be addressed. Firstly, lack of clarification regarding the exact criteria used for the diagnosis of ATLI, and the Hardy-Weinberg equilibrium status among controls in several studies may over inflate our results. Secondly, variation in the anti-TB drugs administered in the studies we analyzed limited the possibility of examining the association of DME with any specific drug. In these studies, multiple anti-TB drugs were utilized in some research, while some other adopts the single-drug therapy. Thirdly, our meta-analysis is based on unadjusted estimates, whereas a more precise analysis could be performed if individual data were available, which would allow for an adjustment estimate (by age, sex, alcohol consumption, cigarette smoking and other lifestyle). Additionally, it should be noted that the studies included patients from several different ethnic groups, with an overrepresentation of East Asian patients (e.g., Chinese, Korean, and Japanese populations) and an underrepresentation of individuals of Caucasian descent. Since allele frequencies may vary considerably between ethnic groups, careful consideration of the potential effect of population genetics on genotypic and phenotypic distribution is warranted, but the limited samples currently available have hampered this effort.

As a result of the heterogeneity of medication used, the duration of illness in different samples, and the different racial groups, it is possible that we have underestimated the effect size of the gene-drug response association. Furthermore, none of the studies formally accounted for medication noncompliance, which is prevalent in patients with TB. Put simply, when a patient does not take the prescribed anti-TB drug, the measured effect size of gene-drug response association is assessed as zero, whereas the true effect of genotype on the phenotype is perhaps larger. Nevertheless, despite the potential underestimation of effect size produced by these uncontrolled factors, we were still able to detect a significant association between the NAT2, GSTM1 and CYP2E1 polymorphism and anti-TB drug response. It is suggested that patients with high-risk genotypes should have regular liver biochemical tests in the first few months following administration of anti-TB drugs. Tailoring the anti-TB regimen to patients according to individual genetic profiles is expected in the coming years.

In summary, our meta-analysis indicates that CYP2E1, NAT2 and GSTM1 genetic variation is significantly associated with anti-tuberculosis drug-induced liver injury. Polymorphisms in these DME, such as NAT2*4, may be particularly important in predicting clinical response to anti-TB drug treatment. Furthermore, interaction between candidate genes and other risk factors, such as diet, alcohol consumption, smoking, existing liver disease and other comorbid diseases, should be explored to realize the modification effect of these extrinsic factors to the expression of different genotypes.

## Supporting Information

Figure S1 **The flow chart of the included studies.**
(TIF)

Figure S2 **Begg’s funnel plot of** ***NAT2*** **polymorphism and ATLI risk.**
(TIF)

Figure S3 **Begg’s funnel plot of** ***CYP2E1*** **polymorphism and ATLI risk.**
(TIF)

Figure S4 **Begg’s funnel plot of** ***GST M1*** **polymorphism and ATLI risk.**
(TIF)

Figure S5 **Begg’s funnel plot of** ***GST T1*** **polymorphism and ATLI risk.**
(TIF)

Checklist S1 (DOC)
