# Supplementary figures and images for: Pharmacogenetic Study of Drug-Metabolising Enzyme Polymorphisms on the Risk of Anti-Tuberculosis Drug-Induced Liver Injury: A Meta-Analysis
Source: PLoS One. 2012 Oct 17;7(10):e47769. doi: 10.1371/journal.pone.0047769 (PMC3474796; doi:10.1371/journal.pone.0047769)

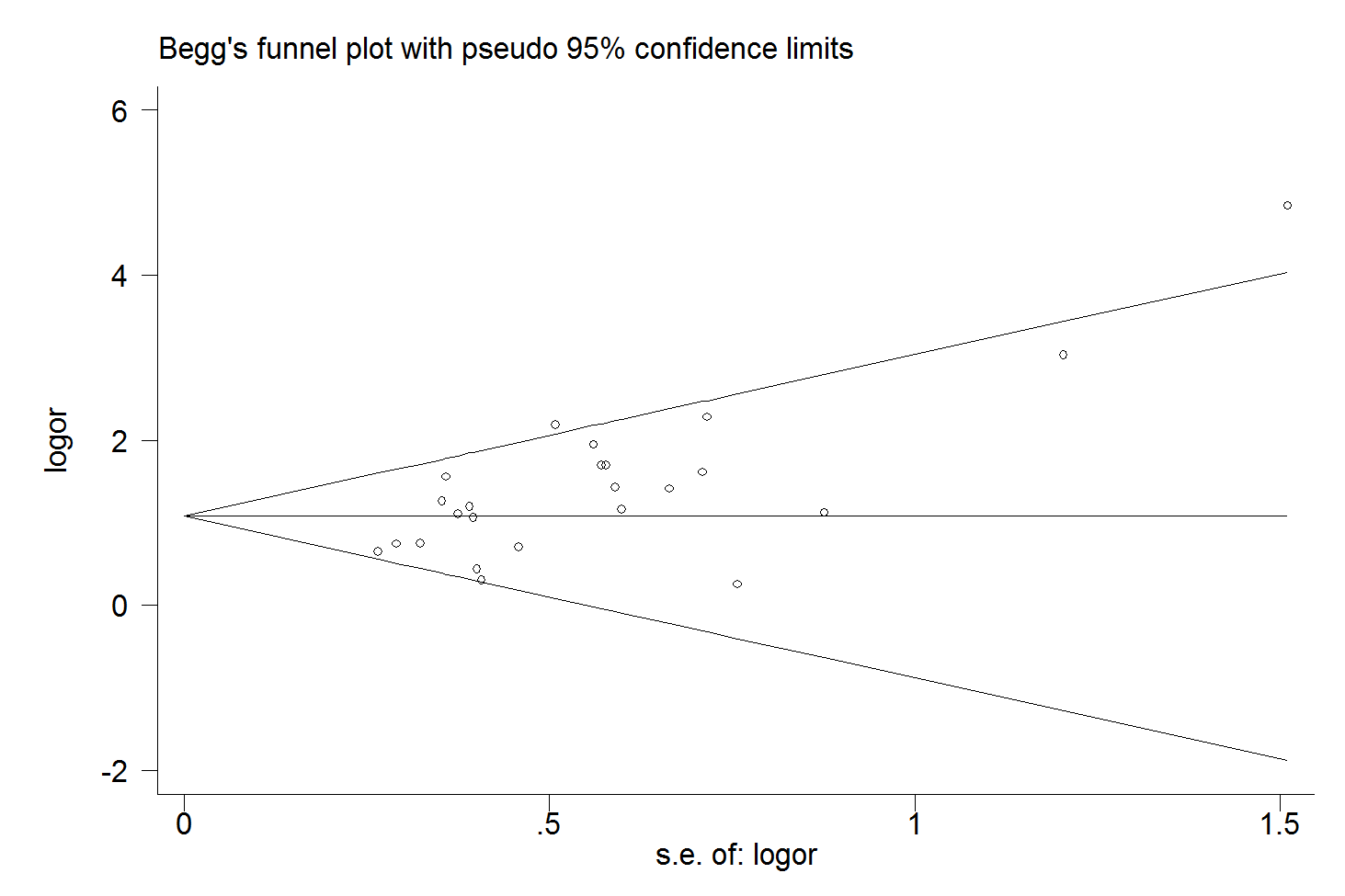

Supplement: Figure S2 — Begg’s funnel plot of NAT2 polymorphism and ATLI risk. (TIF) [file pone.0047769.s002.tif]

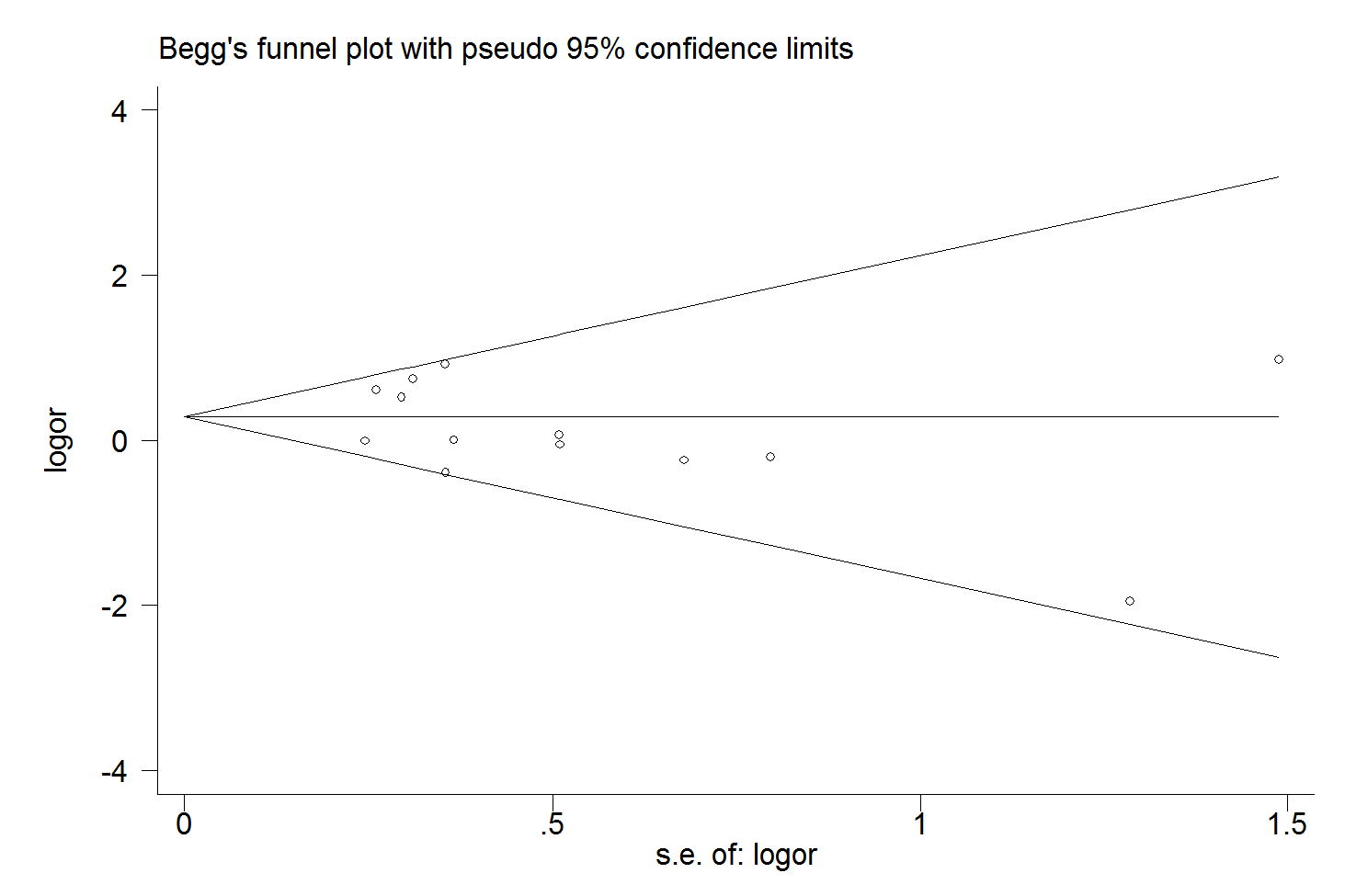

Supplement: Figure S3 — Begg’s funnel plot of CYP2E1 polymorphism and ATLI risk. (TIF) [file pone.0047769.s003.tif]

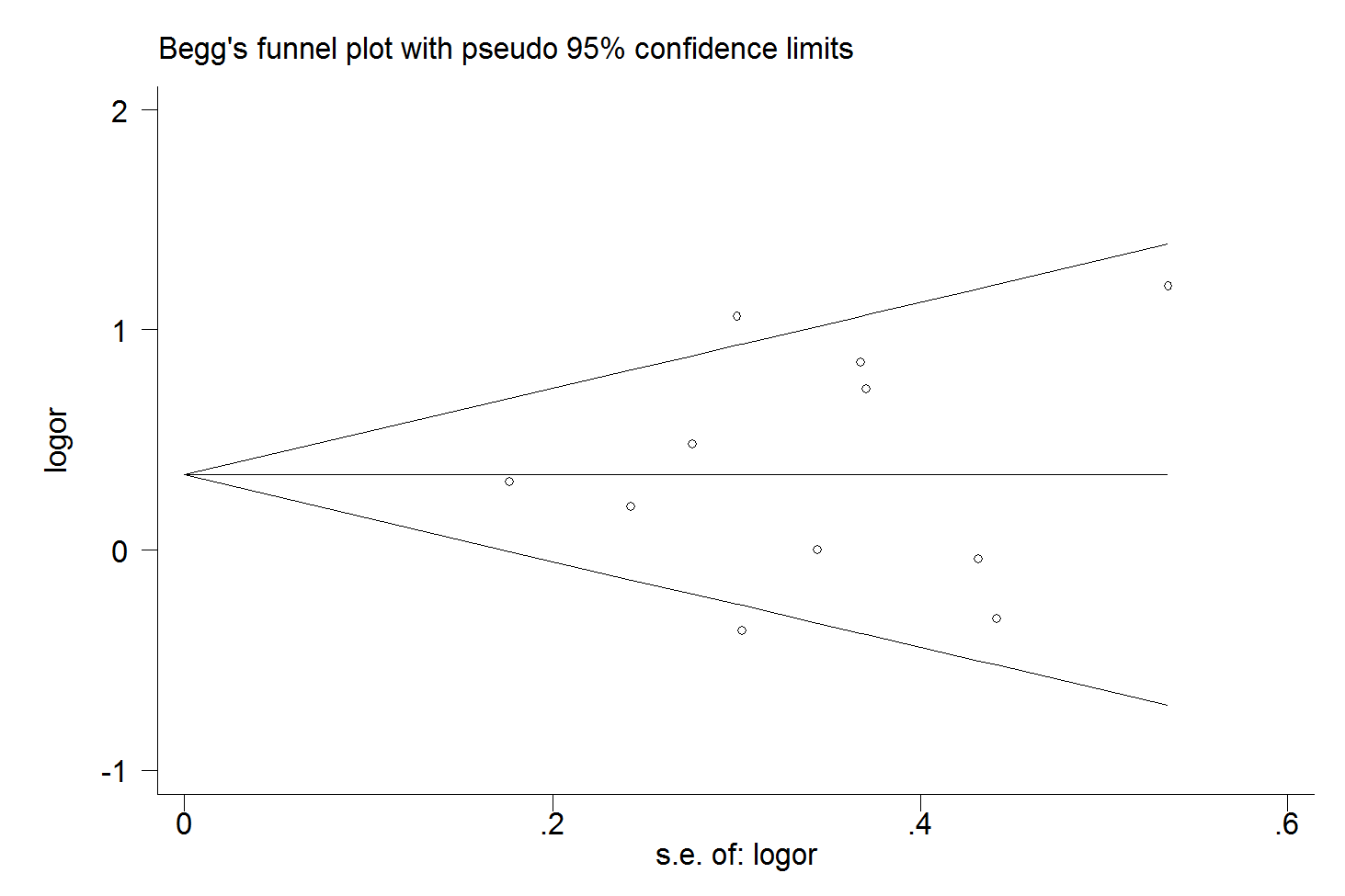

Supplement: Figure S4 — Begg’s funnel plot of GST M1 polymorphism and ATLI risk. (TIF) [file pone.0047769.s004.tif]

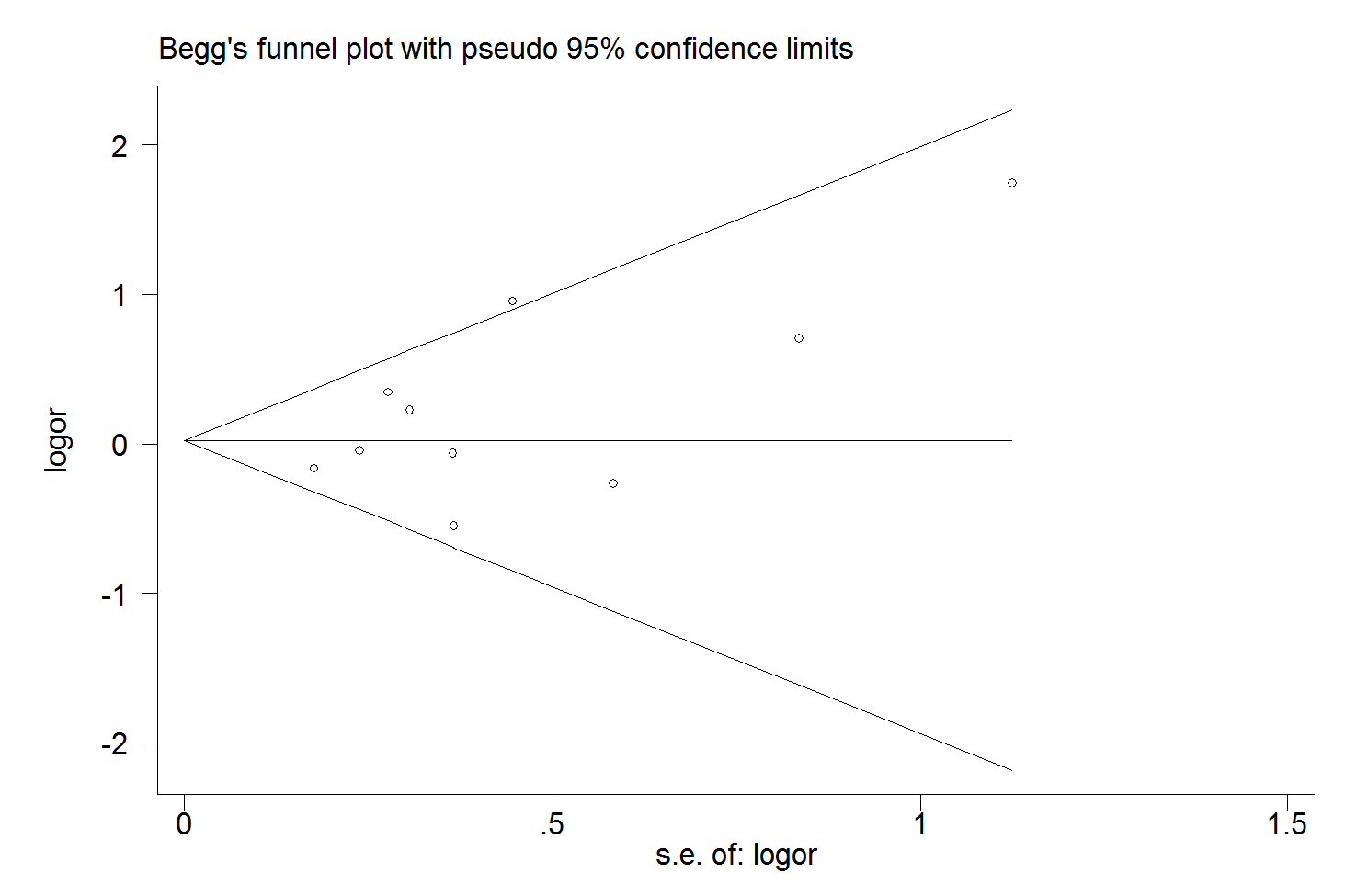

Supplement: Figure S5 — Begg’s funnel plot of GST T1 polymorphism and ATLI risk. (TIF) [file pone.0047769.s005.tif]
